# An Efficient Photomixer Based Slot Fed Terahertz Dielectric Resonator Antenna †

**DOI:** 10.3390/s21030876

**Published:** 2021-01-28

**Authors:** Xiaohang Li, Wenfei Yin, Salam Khamas

**Affiliations:** 1 Department of Electronic and Electrical Engineering, University of Sheffield, Sheffield S10 2TN, UK; s.khamas@sheffield.ac.uk; 2 School of Computer Science and Information Engineering, Hefei University of Technology, Hefei 230009, China; wenfeiyin@hfut.edu.cn

**Keywords:** photomixer, terahertz source, two dimensional photonic crystal, frequency selective surface superstrate, terahertz antenna, dielectric resonator antenna

## Abstract

A slot fed terahertz dielectric resonator antenna driven by an optimized photomixer is proposed, and the interaction of the laser and photomixer is studied. It is demonstrated that in a continuous wave terahertz photomixing scheme, the generated THz power is proportional to the 4th power of the surface electric field of photocondutive layer. Consequently, the optical to THz conversion efficiency of the proposed photomixer has an enhancement factor of 487. This is due to the fact that the surface electric field of the proposed photomixer with a 2D-Photonic Crystal (PhC) superstrate has been improved from 2.1 to 9.9 V/m, which represents a substantial improvement. Moreover, the electrically thick Gallium-Arsenide (GaAs) supporting substrate of the device has been truncated to create a dielectric resonator antenna (DRA) that offers a typical radiation efficiency of more than 90%. By employing a traditional coplanar strip (CPS) biasing network, the matching efficiency has been improved to 24.4%. Therefore, the total efficiency has been considerably improved due to the enhancements in the laser-to-THz conversion, as well as radiation and matching efficiencies. Further, the antenna gain has been improved to 9dBi at the presence of GaAs superstrate. Numerical comparisons show that the proposed antenna can achieve a high gain with relatively smaller dimensions compared with traditional THz antenna with Si lens.

## 1. Introduction

Terahertz (THz) spectrum extends from 300 GHz to 10 THz, which covers the frequency range between mm-wave and infra-red bands. In addition, the corresponding wavelengths represent the transition between photonics and electronics. Higher attention has been paid to the development of THz technologies, owing to the variety of THz spectrum potential applications including monitoring and spectroscopy in pharmaceutical industry [1,2], imaging [3,4], material spectroscopy [5], security [6,7], biology and medicine [8,9], and high-speed communication [10].

However, the main limit to the development of THz technologies is the lack of available THz emitters and detectors [11]. To date, most of the THz systems that utilize time domain techniques employs bulky and expensive femtosecond lasers. In this case, the optical excitation from the lasers can generate and detect sub-picosecond electrical pulses. On the other hand, frequency domain techniques can achieve higher resolutions and high scanning speed in a low cost and portable devices [12]. So far, it has been demonstrated by numerous that continuous wave THz sources can either be generated directly or converted up and down from microwave and optical frequencies, respectively [11]. Nevertheless, there is a lack of efficient room temperature THz sources without the need of cryogenic cooling system and external magnetic field [13].

One of the most promising continuous wave THz sources that utilizes Optical Heterodyne Generation (OHG) [14] is known as photomixer that is capable of generating tunable and coherent THz signals with low-cost and low power consumption in a compact devices [15,16,17,18]. A photomixer consists of two set of metal electrodes, a photoconductive layer and a bulky supporting dielectric substrate. In the photomixer, as two interfering laser beams incident on the non-linear photoconductive medium, electrons are excited from valence band to conduction band, hence, spatiotemporal electrons and holes are generated. Owing to the applied DC biasing voltage, induced photocurrent is driven at the beating frequency of two incident laser beams [19]. Typically, a conventional photomixer can only achieve 0.1% optical to THz power conversion efficiency [20,21]. It has been demonstrated earlier that an enhancement factor of 4 in the optical to THz conversion efficiency can be achieved by using plasmonic material as interdigital electrodes [22]. Additionally, more than 4 times of THz power has been achieved by using optical antenna array of ZnO nanorods [23]. Further, an enhancement factor of 25 in terahertz radiation has been demonstrated by utilizing transparent-conducting oxcides nanocylinders between photomixer electrodes [24]. Additionally, double the effective electric energy can be generated by utilizing embedded electrodes [25]. In addition, to date, a highest reported laser to THz conversion efficiency is 7.5%, where replaced the conventional photomixer electrodes have been replaced by three-dimensional plasmonic contact electrodes [26,27]. However, though many researchers have attempted to optimize the optical to THz conversion efficiency, it is still significantly less than tenth of the theoretical maximum of 100% [28].

As mentioned previously, a photomixer is implemented on a photoconductive layer that is supported by a bulky dielectric substrate. The THz antenna’s input resistance is expected to be reduced by a factor of √((ε_r_ + 1)/2) due to the presence of the bulky dielectric supporting layer that has a dielectric constant of ε_r_. On the other hand, the output resistance of a photomixer is in the order of ~10 kΩ [29]. Therefore, the reduced input resistance of the THz antenna leads to a poorer matching efficiency. A full wavelength dipole has been used to drive a Yagi-Uda array to achieve an input resistance of 2.6 kΩ [30]. Moreover, a 3.3 kΩ input resistance has been achieved by implementing an isolating metallic ground plane with a dipole placed on a thin dielectric slab [31].

In addition, it has been reported that THz communications are more likely to be influenced by the atmosphere, especially, the humidity [32]. Therefore, the radiation power enhancement becomes another challenge. In this case, multiple types of Si lenses have been utilized to achieve a higher gain [33,34]. Beyond that, with the presence of a thick supporting dielectric substrate, Si lens can be used to collect and collimate the generated THz power to minimize the power dissipation in the substrate. However, the usage of Si lens makes the entire antenna configurations even larger on the top of the usage of a thick supporting dielectric substrate.

In this study, the optical to THz conversion efficiency of the photomixer has been optimized based on a numerical study and the utilization of a two-dimensional photonic crystal optical frequency selective surface (FSS) superstrate. Then, a dielectric resonator antenna (DRA) with the appealing features of low cost, small size, and high radiation efficiency, as well as gain [35], has been truncated from the bulky dielectric supporting GaAs substrate, in this case, the low temperature grown GaAs, LT-GaAs, to reduce the size of the antenna configuration and enhances the radiation efficiency. Coplanar stripline and THz dielectric superstrate have been implemented for the further optimization of matching efficiency and antenna gain, respectively. The simulations have been conducted using computer simulation technology (CST) microwave studio.

## 2. Photomixer Design

In this section, the generated THz power from a photomixer, that is biased using a DC voltage and is illuminated by two laser beams with a difference in their central frequencies in the THz range has been calculated analytically. According to the numerical analysis, the generated THz power can be enhanced substantially by optimizing the photomixer configuration.

### 2.1. Derivation of the Generated THz Power from the Photomixer

**Figure 1:**
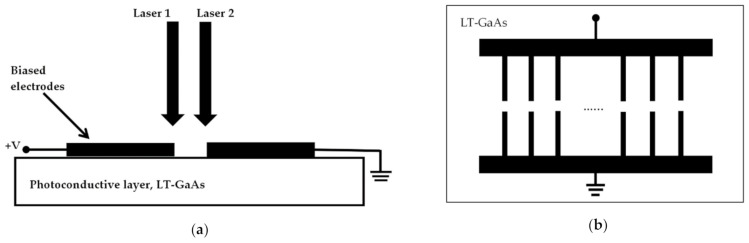
(**a**) Typical photoconductive photomxing scheme; (**b**) Top view of photomixer electrodes.

**Figure 2:**
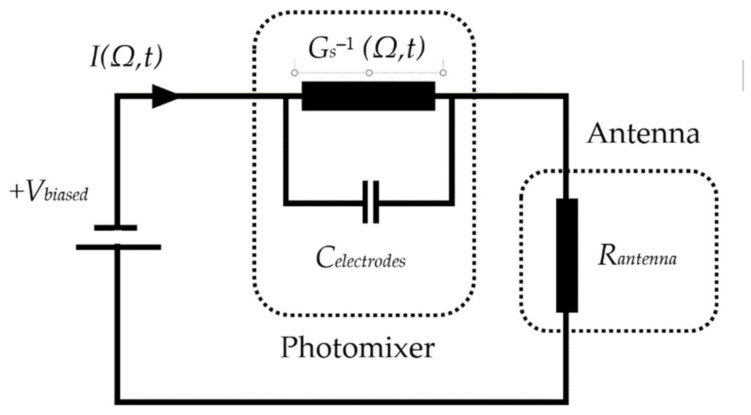
Equivalent circuit of photomixer based THz antenna.

Figure 1a illustrates two linearly polarized continuous wave laser beams with beating frequency falling in the THz spectrum that are incident on the DC biased photoconductive layer. On the other hand, the structure of a typical photomixer electrodes is presented in Figure 1b. Since the applied biased voltage cannot change the absorption coefficient, mobility and recombination time of the low temperature grown GaAs, LT-GaAs photoconductive layer, the electrodes of the photomixer can be considered as an ohmic conductances while the time varying source conductance is electrically modeled as a photoconductance. Consequently, the photomixer based THz antenna can be modeled using the equivalent circuit illustrated in Figure 2 from which it can be noted that the photomixer consists of a photoconductance, *G_s_^−^*^1^*(Ω,t)*, and a paralleled capacitance, *C_electrodes_*. The capacitance depends on the structure of the photomixer and the dielectric constant of the photoconductive layer. The generated photocurrent is driven by the biased voltage excites and the THz radiating antenna, which is represented in Figure 2 by a resistance of *R_antenna_* [36].

The electric fields of the two incident laser beams on the LT-GaAs’ surface can be expressed as:

(1) $$E=\left|E_{i}\right|e^{j\omega_{i}t}$$

where *ω* is the lasers’ angular frequency and *I* = 1,2, represents laser 1 and laser 2, respectively. The laser intensity been absorbed by the LT-GaAs is proportional to the square of the total incident electric field on the LT-GaAs’ surface:

(2) $$I(\Omega ,t)=(1-\Gamma )\sum_{i}{\left|E\right|}^{2}=I_{0}(1-\Gamma )[1+2\frac{\sqrt{mI_{1}I_{2}}}{I_{0}}\cos(\Omega t)]$$

where *I*_0_ is the maximum optical intensity on the LT-GaAs’ surface, *Γ* is the reflection coefficient at the LT-GaAs-air interface, *m* describes the overlap of the laser beams, which is known as the mixing efficiency and *Ω* is angular beat frequency, (*ω*_1_−*ω*_2_).

The induced photo-carriers generated from the incident laser beams as a function of time is:

(3) $$\frac{dn(t)}{dt}=-\frac{n(t)}{\tau_{c}}+\frac{\alpha (T)}{hf_{l}}I(\Omega ,t)$$

in which *h* is the Plank’s constant, *f_l_* is the mean frequency of the laser beams and *τ_c_* is the carrier lifetime. In addition, *α(T)* is the temperature-dependent absorption coefficient and *T* is the temperature in kelvin. For a GaAs layer with a direct band gap, *α(T)* can be expressed as [37]:

(4) $$\alpha (T)\;\approx \;K_{abs}\sqrt{\frac{hf_{l}-E_{g}(T)}{q}}$$

where *K_abs_* is a certain frequency-independent constant which is approximately 9.7 × 10^15^ for GaAs [37], and *E_g_(T)* is the LT-GaAs’ temperature dependent band gap energy defined as:

(5) $$E_{g}(T)=E_{g}(0)-\frac{\alpha_{E}T^{2}}{T+\beta_{E}}$$

in which *E_g_*(0) is the GaAs’ gap energy at 0 °K which is about 1.519 eV, *α_E_* and *β_E_* are material constants of GaAs which are approximately 5.41 × 10^−4^ eV/K and 204 K, respectively [38].

By assuming that *I*_1_ = *I*_2_ = *I*_0_ and *t*/*τ_c_* >> 1, then substituting (2) into (3), the generated carrier density can be obtained as:

(6) $$n(\Omega ,t)=\frac{\alpha (T)}{hf_{1}}I_{0}(1-\Gamma )\tau_{c}(1+\sqrt{m}\frac{\cos(\Omega t)+\Omega \tau_{c}\sin(\Omega t)}{1+{\left(\Omega \tau_{c}\right)}^{2}})$$

The conductance of the photomixer can be expressed as:

(7) $$G_{s}(t)=\int dG_{s}(t)=\int_{0}^{T_{sub}}\sigma (t)e^{-\alpha (T)z}\frac{W}{L}dz=\frac{W}{\alpha (T)L}\sigma (t)(1-e^{-\alpha (T)T_{\mathrm{sub}}})$$

in which *T_sub_* is the depth of photoconductive region, *W* is the width of the electrode, *L* is the length of the electrode and *σ(t)* is the conductivity. The electrical conductivity is defined as:

(8) $$\sigma (t)=e\mu_{e}n(t)=\frac{\alpha (T)e\mu_{e}}{hf_{l}}I_{0}(1-\Gamma )\tau_{c}(1+\sqrt{m}\frac{\cos(\Omega t)+\Omega \tau_{c}\sin(\Omega t)}{1+{\left(\Omega \tau_{c}\right)}^{2}})$$

where *e* is the electron charge and *µ_e_* is the electron mobility. Therefore, the photomixer’s conductance can be derived by substituting (8) into (7):

(9) $$G_{s}(\Omega ,t)=\frac{We\mu_{e}I_{0}\tau_{c}}{hLf_{l}}(1-\Gamma )(1{-e}^{-\alpha {(T)T}_{\mathrm{sub}}})(1+\sqrt{m}\frac{\cos(\Omega t)+\Omega \tau_{c}\sin(\Omega t)}{1+{\left(\Omega \tau_{c}\right)}^{2}})$$

**Figure 3:**
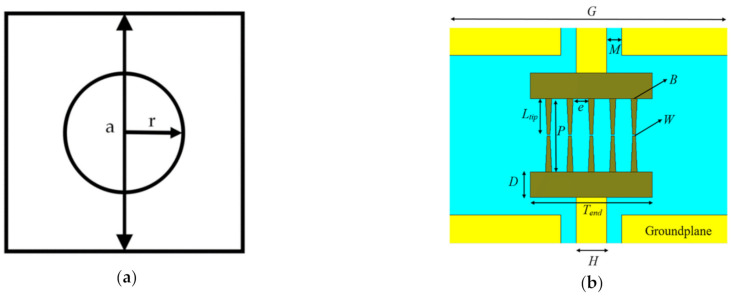
(**a**) Configurations of the 2D-PhC unit cell; (**b**) Top view of the photomixer based slot.

The impedance of the system can be given by analyzing the equivalent circuit shown in the Figure 3:

(10) $$Z_{t}(\Omega ,t)=\frac{1}{j\Omega C_{electrodes}+G_{s}(\Omega ,t)}+R_{antenna}$$

and the radiation power can be defined as:

(11) $$P_{THz}(\Omega ,t)=R_{antenna}{\left(\frac{V_{biased}}{Z_{t}(\Omega ,t)}\right)}^{2}$$

Therefore, as *R_antenna_G_s_* is much smaller than 1 and by replacing system impedance by (8), as well as neglecting the imaginary part, the radiation power can be expressed as:

(12) $$P_{THz}(\Omega ,t)\;\approx \;R_{antenna}\frac{V_{biased}^{2}G_{s}^{2}(\Omega ,t)}{1+{\left(\Omega R_{antenna}C_{electrodes}\right)}^{2}}$$

In addition, by employing (9) and averaging the power, the mean generated THz power can be expressed as:

(13) $$P_{THz}\approx {\left[\frac{We\mu_{e}\tau_{c}}{hLf_{l}}(1-\Gamma )(1{-e}^{-\alpha {(T)T}_{\mathrm{sub}}})\right]}^{2}(\frac{mR_{antenna}V_{biased}^{2}}{\left[1+{\left(\Omega R_{antenna}C_{electrodes}\right)}^{2}][1+{\left(\Omega \tau_{c}\right)}^{2}\right]})I_{0}^{2}$$

It can be noted from (13) that the generated THz power depends on three main factors, (*Ω R_antenna_ C_electrode_*), (*Ωτ_c_*) and *I_0_*^2^. As mentioned previously, *R_antenna_* and *C_electrodes_* depend on the photomixer’s configuration and dielectric constant of the photoconductive layer. In addition the carrier lifetime is a function of the applied bias voltage [38,39,40]. Hence, it can be demonstrated that for the same photoconductive material and photomixer configuration that are used with the same biased voltage, the generated THz power is proportional to the square of the incident laser intensity on the surface of the LT-GaAs layer. Since the intensity is proportional to the square of electric field, the generated THz power is proportional to the 4th power of the electric field at the surface of LT-GaAs. Consequently, a design that optimizes the laser intensity is proposed instead of manipulating the photoconductive material and photomixer electrodes’ configuration.

### 2.2. Photomixer Modeling

In order to optimize the electric field on the surface of LT-GaAs, two dimensional photonic crystal (2D-PhC) has been introduced by utilizing a periodic plane and a non-periodic third dimension to provide a pass, or stop, band frequency response [41]. The unit cell of the 2D-PhC is illustrated in Figure 3a. The 2D-PhC has been used as an optical frequency selective surface (FSS) superstrate that is placed at an optimum height above the photomixer. The electromagnetic wave bounces between the FSS and ground plane surrounding the photomixer, therefore, the cavity created by FSS superstrate and ground plane can enhance the optical intensity on the surface of the LT-GaAs.

**Figure 4:**
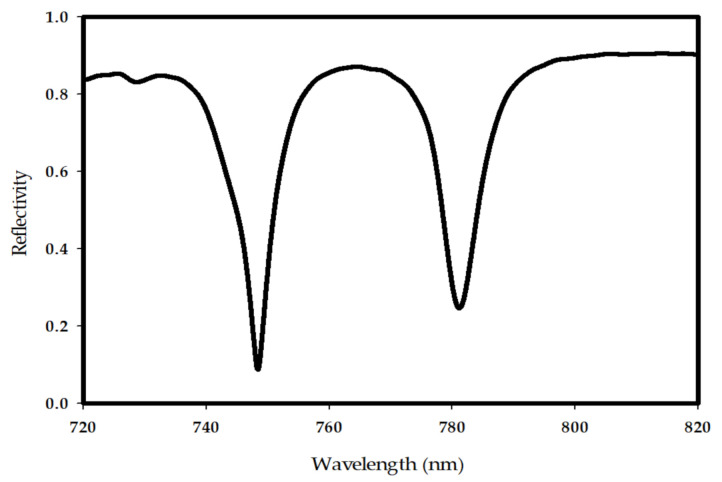
Reflectivity of the 2D-PhC FSS.

Since the photomixer is used as a source to excite the truncated GaAs THz DRA, the metallic ground plane has been deployed on top of the LT-GaAs photoconductive layer. However, in order to illuminate the photomixer by the laser beams, a central slot feed is used to accommodates the photomixer as shown in Figure 3b. Moreover, the electrodes have been defined as optical gold (Palik) for CST simulation purposes. The dimensions of the parameters shown in Figure 3b have been defined as: *M* = 0.5 µm, *B* = 0.2 µm, *W* = 0.1 µm, *e* = 0.5 µm, *P* = 2.3 µm, *L* = 1.13 µm, *D* = 0.8 µm, *T_end_* = 4 µm, *H* = 1 µm, *G* = 13.68 µm, and thickness of 0.1 µm. It should be noted that the thickness of the LT-GaAs photoconductive layer is 0.44 µm with a relative dielectric constant of 12.9. The material of the 2D-PhC FSS has been assumed as GaAs while the periodicity, central air hole radius, and thickness have been chosen as *a* = 0.76 µm, *r* = 0.3 *a* and *h* = 0.2 *a*, respectively. Figure 4 illustrates the reflectivity of the 2D-PhC FSS, where it can be noted that there is a stopband at wavelength range of 750 to 780 nm.

**Figure 5:**
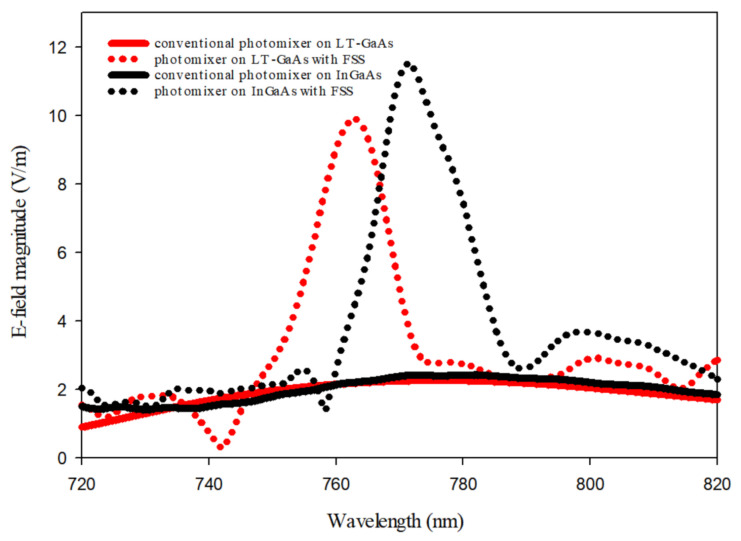
Optical E-field magnitude between the central electrodes of a photomixer on the surfaces of LT-GaAs and I GaAs photoconductive layers.

**Figure 6:**
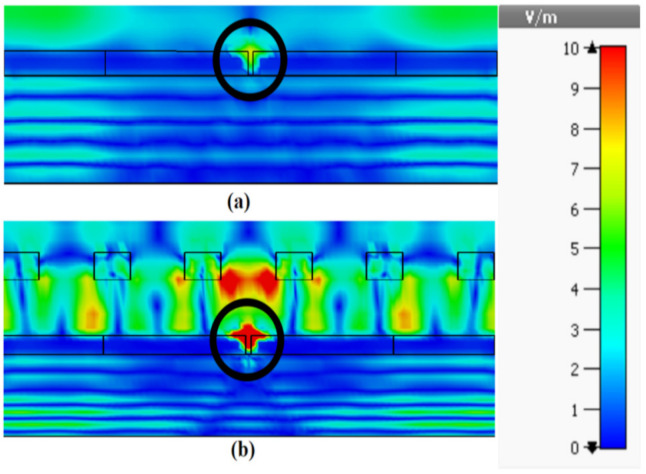
The optical E-field distribution (**a**) without FSS (**b**) with FSS.

A 2D-PhC layer with 19 × 19 unit cells has been suspended at a height of 0.3 µm above the photomixer to act as an FSS superstrate. The incident laser beams have been modeled as a linearly polarized plane wave with a 1 V/m electric field component along the direction of photomixer electrodes. The electric field magnitudes between the central electrode pair on the LT-GaAs’ surface is illustrated in Figure 5 with the comparison to that at the absence of 2D-PhC FSS superstrate. In addition, the cross-section of the surface electric field distribution at the central pair of photomixer electrodes are presented in Figure 6. From these results, it can be observed that the utilization of the 2D-PhC superstrate has improved the electric field on the electrodes from 2.1 to 9.9 V/m, which represents an enhancement factor of 4.7. As explained earlier, the generated THz power is proportional to the 4th power of the electric field, therefore the corresponding enhancement factor of the generated THz power is 487. Besides, the same methodology has been applied to an identical photomixer albeit with a InGaAs photoconductive layer, where the electric field on the InGaAs’s surface has increased form 2.42 to 11.5 V/m by utilizing an FSS superstrate with unit cell’s dimension of *a* = 0.72 µm *r* = 0.27 *a* and *h* = 0.19 *a* at a height of 0.23 µm above the photomixer. The results are presented in Figure 5, where it can be noted that approximately same enhancement factor has been achieved compared to the LT-Gaas photoconductive layer. Compared with the cases of increasing the electrodes E-field from 2.1 to 3.4 V/m and 4.365 V/m using 2D-PhC, with central hole [42] and plasmonic rod [43], respectively, as reflectors underneath the photoconductive layer, employing a 2D-PhC as FSS superstrate has substantially enhanced the optical to THz power conversion efficiency. However, the overall efficiency of the system depends on the laser to THz power conversion efficiency as well as the antenna’s radiation and matching efficiencies that will be investigated next. In the following section, the optimized photomixer will be used to excite a THz DRA that is truncated from the supporting bulky GaAs substrate. A DRA has been chosen due to the high radiation efficiency of more than 90% at the frequency range of interest.

## 3. THz Dielectric Resonator Antenna Design

### 3.1. Antenna Configuration

The presence of the bulky GaAs supporting substrate reduces the input impedance and absorbs most of the generated THz power, which impairs the matching and radiation efficiencies. A typical radiation efficiency for a dipole above a thick dielectric substrate is 40% or less depending on the thickness and dielectric constant of the substrate [44]. On the other hand, a rectangular dielectric resonator antenna offers a considerable enhancement in the radiation efficiency owing to the absence of surface waves and ohmic losses. Therefore, the bulky GaAs substrate can be truncated to act as a dielectric resonator antenna that operates at the higher order resonance mode. However, the truncated DRA should be large enough to maintain the required physical support to the photomixer device. For fabrication purposes, the width to height aspect ratio of the truncated DRA should be greater than 3. Otherwise, a fragile configuration will be achieved that is difficult to fabricate. Furthermore, the utilization of a DRA as a substrate results in a much smaller configuration compared to traditional structures that are based on utilizing a hemispherical Si lens to extract the THz power. As the configurations of the photomixer and corresponding 2D-PhC FSS superstrate are relatively small enough at the THz spectrum, they will have a negligible impact on the performance at the THz frequency range.

**Figure 7:**
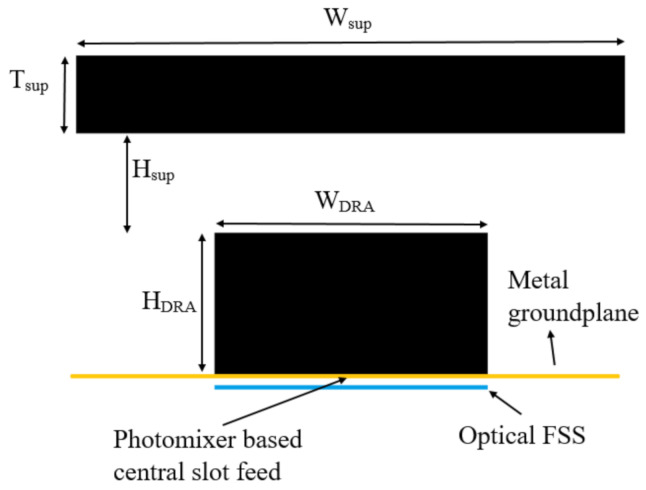
THz DRA and superstrate configuration.

As a result, the GaAs substrate has been employed as the THz antenna that also provides the mechanical support to the device at the same time. The GaAs DRA is illustrated in Figure 7 with dimensions of *W_DRA_* = 250 µm, *H_DRA_* = 60 µm, as well as a relative dielectric constant of 12.9, and has been placed on a gold ground plane with a size of *W_ground_* = *W_sup_* = 400 µm. For further gain enhancement, an additional GaAs dielectric superstrate has been employed with dimensions of *W_sup_* = 400 µm and *T_sup_* = 60 µm. As illustrated in Figure 7, the original optical superstrate has been placed on the feed side of the DRA to capture the illuminating laser beams, while this THz superstrate is placed above the opposite side of the DRA to enhance the radiated THz power. Therefore, the two superstrates will not impact each other as they separated by the DRA and the gold ground plane that accommodates the photomixer. The distance between the DRA and the new THz superstrate can be determined as *H_sup_* = (0.25*((*φ*_1_ + *φ*_2_)/*π*) + 0.5) *λ* [45], where *φ_1_*, *φ_2_* represents the reflection coefficient phases of the superstrate and ground plane. Therefore, the distance between the GaAs DRA and the THz superstrate has been calculated as *H_sup_* = 30 µm.

**Figure 8:**
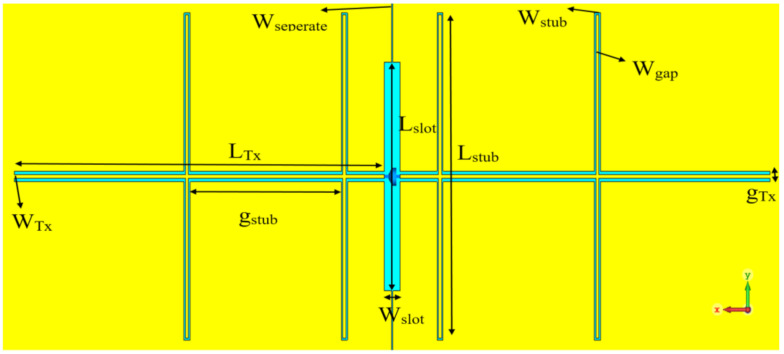
Top view of feeding slot and CPS.

DC bias is required to generate the THz power, therefore, the ground plane has been divided into two halves by a narrow slot with a width of W_seperate_ = 0.5 µm to work as two large DC biasing pads as illustrated in Figure 8. Since the generated THz power can leak through the DC biased pads and transmission line, a coplanar stripline (CPS) network has been employed to work as a choke filter to minimize the THz current leakage, as well as improving the matching. The configuration of the CPS and feeding slot, which accommodates the photomixer and excites the DRA, has been included in Figure 8. The feeding slot has a length of *L_slot_* = 65 µm and width of *W_slot_* = 5 µm. The dimensions of the CPS network have been chosen as *L_Tx_* = 120 µm, *W_Tx_* = 1 µm, *L_stub_* = 91 µm, *W_stub_* = 0.5 µm, *g_stub_* = 50 µm, *W_gap_* = 0.5 µm, and *g_Tx_* = 3 µm, respectively.

Finally, the feeding photomixer has been modeled as a discrete port with a 10 kΩ input resistance that is in parallel with a 3fF lumped capacitance. Both of the discrete port and lumped capacitance have been deployed at the center of feeding slot in order to be connected with the CPS and DC bias pads.

### 3.2. Results and Discussion

**Figure 9:**
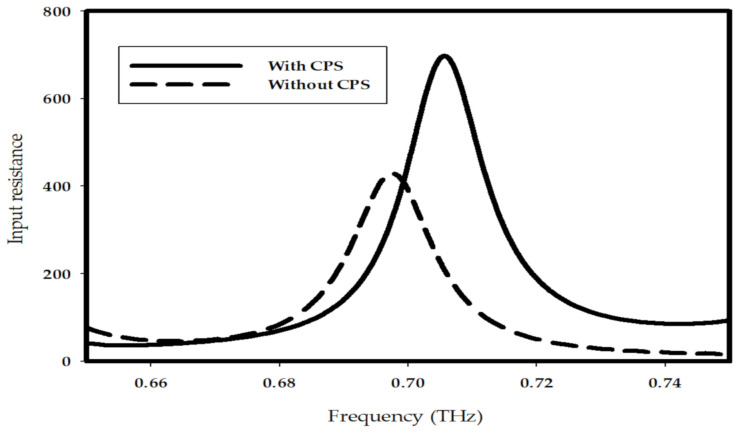
Input resistance of photomixer based slot fed THz DRA with or without CPS.

**Figure 10:**
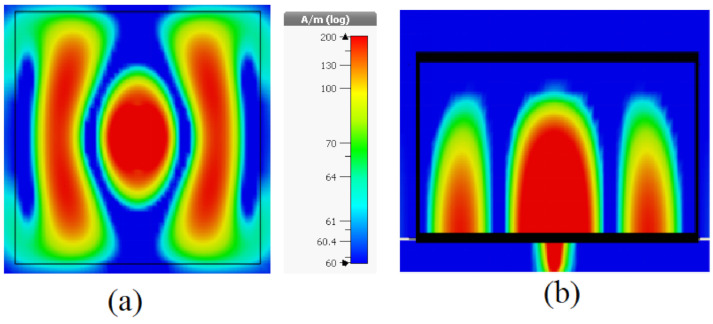
The H-field component inside the DRA with TE_711_mode at (**a**) XY plane; (**b**) XZ plane.

**Figure 11:**
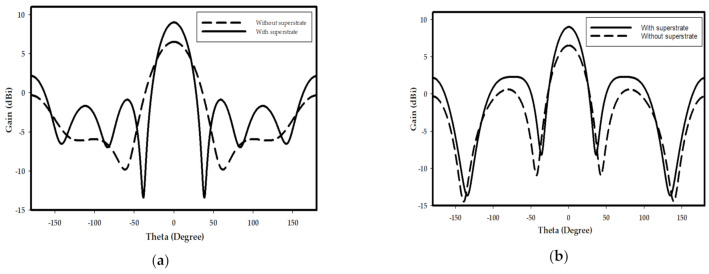
Radiation pattern of DRA with and without superstrate at (**a**) φ = 0°; (**b**) φ = 90°.

The input impedance of the DRA with and without CPS network has been studied as shown in Figure 9, where it can be noted that the input resistance has been improved from 430 to 700 Ω by utilizing the CPS, which corresponding to an enhancement of matching efficiency from 15.8 to 24.5%. Furthermore, the resonance mode of the DRA has been investigated as illustrated in Figure 10, where it can be noted that the TE_711_ mode has been excited. The radiation patterns of the DRA are presented in Figure 11, where the broadside gain has been improved from 6.5 to 9 dBi by incorporating the THz GaAs superstrate. As a result, the radiated THz power has been enhanced by a factor of 2. Therefore, the performance of the THz photomixer based antenna has been improved considerably by combining several factors such as the improving the optical to THz power conversion efficiency as well as enhancing the radiation efficiency by utilizing a DRA and employing a CPS that improved the matching efficiency.

Table 1 compares the performance of the proposed DRA with published THz antennas and THz DRAs, where it can be observed that presented DRA offers a higher antenna gain as compared with other DRAs though, it is slightly larger than the reported DRAs. Compared with other antenna types, the proposed THz DRA achieves a similar gain with much smaller dimensions. Such a miniaturized high gain antenna can be used to optimize the performance of any THz application with a limited system space.

## 4. Conclusions

The presented work introduces a photomixer based slot fed terahertz dielectric resonator antenna with enhanced optical to THz power conversion, as well as improved matching and radiation efficiencies. The interaction of two incident continuous wave laser beams and photomixer has been studied. A general analytical expression for the generated THz power has been derived, which demonstrates that the generated THz power is proportional to the 4th power of the electric field on the surface of photoconductive layer. Therefore, by utilizing a 2D-PhC as FSS, the optical to THz conversion efficiency has been improved by a factor of 487. Consequently, the optimized photomixer has been accommodated in a central slot, which has been used to excite the THz DRA that has been truncated from the thick supporting GaAs substrate. A coplanar stripline has been implemented to minimize the leakage of THz power through DC bias pads and transmission line, as well as improving the input resistance of the antenna. As a result, the input resistance of the DRA has been improved from 430 to 700 Ω, which corresponds to 15.8 and 24.4% matching efficiency, respectively. Finally, a THz GaAs superstrate has been employed with the THz DRA, which leads to an enhancement of the antenna gain from 6.5 to 9 dBi. The presented results demonstrate that the proposed design outperforms other counterparts reported in the literature. As demonstrated by (13), further enhancement of the optical to THz conversion efficiency can be achieved by reducing the carrier lifetime of the photoconductive layer such as changing the configuration of the photomixer electrodes to minimize the traveling distance of carriers. The performance can be improved further by altering the shape and dimensions of the slot to increase the order of the excited mode as this has the potential of providing higher gain. In addition the packaging of the proposed configuration needs to be considered so that a physical support is provided to the Thz superstrate in conjunction with improving the handling and stability of the device.

## Figures and Tables

**Table 1:** Performance summary and comparison with prior works.

Reference	[34]	[46]	[47]	[48]	This Work
Antenna Type	Small lens with Leaky-wave Slit Dipole Antenna	Dipole Antenna with Horn Cavity	Slot Fed stacked DRA	Patch Fed Higher Order Mode DRA	Slot Fed GaAs Substrate Truncated DRA
Frequency (THz)	0.2	1	0.13	0.34	0.7
Antenna Gain	10.3	9.07	4.7	7.9	9
DR Material/ε_r_	-	-	Alumina/10	Silicon/11.9	GaAs/12.9
DR type	-	-	Rectangular	Rectangular	Rectangular
Antenna Aperture (λ^2^)	1.44	4.55	0.72	0.2	0.87
Antenna Height (λ)	1.2	1.16	1.28	0.5	0.35

## Data Availability

Data is contained within the article or supplementary material.
